# Non-Invasive Biometrics and Machine Learning Modeling to Obtain Sensory and Emotional Responses from Panelists during Entomophagy

**DOI:** 10.3390/foods9070903

**Published:** 2020-07-09

**Authors:** Sigfredo Fuentes, Yin Y. Wong, Claudia Gonzalez Viejo

**Affiliations:** Digital Agriculture, Food and Wine Sciences Group. School of Agriculture and Food, Faculty of Veterinary and Agricultural Sciences, University of Melbourne, Parkville, VIC 3010, Australia; awongyinying@gmail.com (Y.Y.W.); cgonzalez2@unimelb.edu.au (C.G.V.)

**Keywords:** entomophagy, neophobia, alternative protein source, emotions, emojis

## Abstract

Insect-based food products offer a more sustainable and environmentally friendly source of protein compared to plant and animal proteins. Entomophagy is less familiar for Non-Asian cultural backgrounds and is associated with emotions such as disgust and anger, which is the basis of neophobia towards these products. Tradicional sensory evaluation may offer some insights about the liking, visual, aroma, and tasting appreciation, and purchase intention of insect-based food products. However, more robust methods are required to assess these complex interactions with the emotional and subconscious responses related to cultural background. This study focused on the sensory and biometric responses of consumers towards insect-based food snacks and machine learning modeling. Results showed higher liking and emotional responses for those samples containing insects as ingredients (not visible) and with no insects. A lower liking and negative emotional responses were related to samples showing the insects. Artificial neural network models to assess liking based on biometric responses showed high accuracy for different cultures (>92%). A general model for all cultures with an 89% accuracy was also achieved.

## 1. Introduction

Increasing public concerns related to the environmental impacts and health-related issues associated with foods from animal sources have influenced the increased research interest in the use and acceptability of alternative protein sources. Insects have been shown as a feasible, sustainable protein source in recent studies [1,2,3,4,5]. The latter is because, for animals, the production of 1 kg of animal weight will typically require around 2.5 kg of feed for chicken, 5 kg for pork, and 10 kg for beef. On the contrary, for insects, to produce the same weight of crickets (*Gryllidae*), around 1.7 kg of feed is required. Furthermore, edible insect parts are around 80% compared to only 55% for chicken and 40% for cattle [6]. Most insects, when compared to plants, have higher protein content—for example, insects have, in general, 35% to 77% protein content per edible weight, compared to soybeans with approximately 35%. Insects are considered as a complete protein food due to the presence and amount levels of all, or most of, the essential amino acids required for adequate human health that cannot be found from plant sources, such as cereals and legumes [7,8]. From an environmental point of view, insects can produce between 88% (cockroaches) and 46% less CO_2_ (crickets and beetles) compared to beef cattle per unit of weight gain. Furthermore, the production of other greenhouse gases with 300 and 84 times more potency compared to CO_2_, such as N_2_O and CH_4_, respectively, are negligible compared to those produced by beef cattle or pigs [9].

Although the consumption of insects dates from ancient times and up to 100 million years ago from anthropological studies, and they are currently a common source of food in more than 100 countries [10,11], most cultures are still reluctant to include them as part of their daily diets [3]. In previous studies, food neophobia and disgust have been reported as the main reasons for consumers being hesitant to try insect-based foods, especially for Westerners [1]. Other studies have reported that curiosity, novelty, and interest in searching for healthier meat alternatives are the main drives for consumers to try insect-based foods [12,13]. It has also been found that consumers are more willing to try these foods when the insects are used as part of their ingredients and are not visible, compared to when they can see the whole insect or parts of them within the presented dish [1,14]. Therefore, the initial assessment of the visual attributes of dishes of insect-based foods is very important, as they create the first impression for consumers and determine their eagerness to taste the product or not. Furthermore, the presentation of food, beverages, and even the packaging of food products using imagery presented on digital screens renders statistically similar information when presenting the same product for taste or handling, as it creates the first impression for consumers when judging a product [15,16,17]. Hence, the visual renderings of insect-based food may help to break negative emotions related to first impressions.

Due to the influence of emotions in decision-making, especially for new food products and even food packaging for consumer acceptability [15,16,18,19,20,21], it is important to assess how insect-based food products make consumers feel, besides studying only their acceptability. Therefore, some studies have been conducted to evaluate emotional responses towards foods with insects [22], specifically focused on disgust [23,24]. More recently, the use of emojis has been implemented for this purpose, as consumers tend to identify emotions using proxy images related to expressed emotions as non-verbal cues, as these are more closely associated with their feelings [25,26,27]. Subconscious responses from consumers have also been evaluated for chocolate and beer using computer vision techniques and machine learning to assess their facial expressions [15,16,20,28,29]. These studies may produce more relevant information that may be missed using conventional self-reported sensory analysis, especially for insect-based food products.

Therefore, this study aimed to assess consumers’ self-reported and subconscious emotional responses towards different insect-based food samples evaluated using emojis, video capturing, and analysis (biometrics) as a quick and reliable approach to understand emotional responses according to cultural backgrounds. To achieve this, a cross-cultural analysis was conducted to compare the responses of Chinese and Australian participants. A sensory session was conducted using the BioSensory application (app; University of Melbourne, Melbourne, Vic, Australia; [30]) to obtain biometrics and check all that apply (CATA), using emojis to assess emotions and a 15 cm non-structured scale to evaluate the acceptability of five different foods prepared with insects. Furthermore, machine learning (ML) based on artificial neural networks (ANN) models were developed to classify samples into low and high overall liking using the subconscious (biometrics) emotional responses as inputs.

## 2. Materials and Methods

### 2.1. Sensory Session Description and Video Analysis

A total of 88 participants (34 Asians and 54 non-Asians) were recruited from the pool of staff and students at the University of Melbourne (UoM), Australia. The Asian participants were from countries such as China, Malaysia, Vietnam, Philippines, India, and Indonesia, while the non-Asians were from Australia, New Zealand, Mexico, Colombia, Germany, Ukraine, and the United States of America. The ethics considerations were related to the minimal risks associated with personal information and image/video acquisition from participants. Ethics approval was granted by the Faculty of Veterinary and Agricultural Sciences (FVAS) from the UoM with ID: 1545786.2. Furthermore, proper data handling and storage have been followed for security reasons and will be stored for five years. The participants were asked to sign a consent form for them to allow being video-recorded and to specify any allergies to assess whether they may take the test. The only information that was provided to participants about the samples along with the consent form previous to the test was “Some samples may contain insects”, due to the allergic reactions that these may cause. A power analysis was performed using SAS^®^ Power and Sample Size version 14.1 software (SAS Institute Inc., Cary, NC, USA), showing that the number of participants per culture was sufficient to find significant differences among the samples and cultures (Power: 1 − β > 0.99). The session was conducted in individual sensory booths located in the sensory laboratory belonging to the FVAS of the UoM (Melbourne, Australia) using the BioSensory app [30] to display the questionnaire and automatically record videos of the participants while assessing the insect-based food samples. The participants were asked to taste two control and three different insect-based food samples (Table 1), one at a time, and rate their liking for different descriptors as well as to indicate how they felt towards the different samples using a FaceScale (FS; Table 2) and check all that apply (CATA) tests for different emojis that best represented their emotions towards the sample (Table 3). The samples were assigned a 3-digit random number and served at room temperature (20 °C); the avocado was prepared just before serving, and drops of lime juice were added to avoid oxidation and darkening. The participants were provided with water and water crackers as palate cleansers between samples.

Videos were recorded along with the self-reported answers from participants using the BioSensory app and analyzed using a second app developed with the Affectiva software development kit (SDK; Affectiva, Boston, MA, USA). The latter app can analyze videos in batch to obtain the facial expressions from participants based on the micro- and macro-movements of the different features of the face, using the histogram of the oriented gradient for computer vision analysis. Additionally, it uses machine learning algorithms based on support vector machine to translate facial expressions into emotions and emojis; it also assesses head movements (Table 4).

### 2.2. Statistical Analysis and Machine Learning Modeling

An ANOVA was conducted for the quantitative self-reported and biometric responses with a Fisher’s least significant differences (LSD) *post hoc* test to assess significant differences (α = 0.05) between samples nested within cultures (Asians and non-Asians) using XLSTAT ver. 2020.3.1 (Addinsoft, New York, NY, USA). Furthermore, multivariate data analysis was conducted using principal component analysis (PCA) to find the relationships and associations among samples and variables from the quantitative self-reported and biometric responses using Matlab^®^ R2020a (Mathworks, Inc., Natick, MA, USA). On the other hand, to find relationships between the frequency responses from emojis using the CATA test and quantitative self-reported and biometric responses as well as the associations of samples with each response, a multiple factor analysis (MFA) was conducted using XLSTAT. Furthermore, a correlation matrix was developed using Matlab^®^ R2020a to assess only the significant correlations (*p* ≤ 0.05) between the quantitative self-reported and biometric responses.

**Figure 1:**
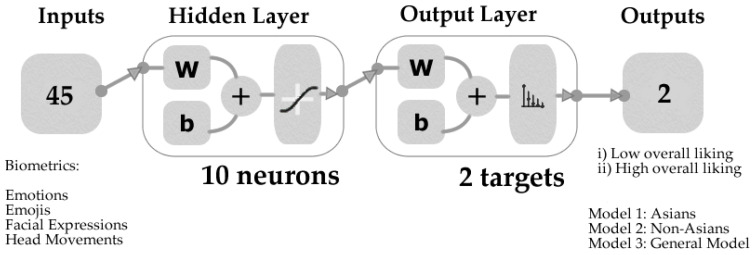
Diagram of the artificial neural network two-layer feed-forward models showing the number of inputs (Table 4), the outputs/targets, and the number of neurons.

The machine learning (ML) models based on artificial neural networks (ANN) pattern recognition were developed using a code written in Matlab^®^ R2020a to evaluate 17 different training algorithms. Bayesian Regularization was selected as the best algorithm, resulting in models with higher accuracy and no signs of overfitting from performance tests. A total of 45 inputs from the biometrics emotion analysis (Table 4) were used to classify the samples into low and high overall liking. Model 1 was developed using data from Asian participants, while Model 2 was constructed using data from non-Asians. A further ML model (Model 3) was created as a general model, using the results from all the participants regardless of their cultural background. The data were divided randomly using 80% of the samples (samples x participants) for training and 20% for testing. The performance assessment was based on the mean squared error (MSE), and a neuron trimming test (3, 7, 10 neurons) was conducted to find the models with the best performance and no overfitting from performance tests. Figure 1 shows the diagram of the ANN models developed using a tan-sigmoid function in the hidden layer and Softmax neurons in the output layer.

## 3. Results

### 3.1. Results from the ANOVA of Self-Reported and Biometric Responses

Results from the ANOVA of the self-reported responses showed that there were significant differences between samples for the eight descriptors considered (Table S1). However, non-significant differences were found between the cultures for appearance, FS App, aroma, texture, and overall liking. For flavor, overall liking, and purchase intention, there were significant differences between cultures for the tortilla chips made with cricket flour and the avocado toast with crickets. Likewise, for FS Taste, there were significant differences between cultures for the avocado toast with crickets. The control samples of tortilla chips and avocado toast, along with the tortilla chips made with cricket flour, were the most liked in terms of appearance and texture. For Asians, besides the control samples, the tortilla chips made with cricket flour were the most liked in terms of texture and overall but were significantly different from the control. Conversely, for non-Asians, the tortilla chips made with cricket flour were amongst the highest in liking of all sensory descriptors as well as in the FaceScale rating, and non-significant differences were found with the control samples. Similarly, for non-Asians, the avocado toast with crickets was amongst the highest in flavor liking, with non-significant differences compared to the control samples.

The results of the ANOVA for the biometrics responses showed there were significant differences between the samples and/or cultures (Table S2). There were significant differences between the cultures for head roll 
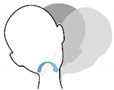
 when assessing the avocado toast with crickets and the whole crickets, with more negative results for the Asians, which means that they moved the head to the right side when evaluating these samples. For joy, there were significant differences between cultures, with the avocado toast control sample having higher expression of this emotion in Asians. The Asians also behaved significantly differently to the non-Asians in terms of engagement when assessing the tortilla chips made with cricket flour, with the Asians being more engaged with the sample. The Asians expressed significantly more smiley faces 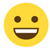 when evaluating both the avocado toast control and the toast with crickets than the non-Asians. Likewise, the Asians expressed significantly more stuck out tongue with winking eye 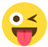 expressions when assessing the tortilla chips made with cricket flour.

### 3.2. Multivariate Data Analysis

#### 3.2.1. Principal Components Analysis

**Figure 2:**
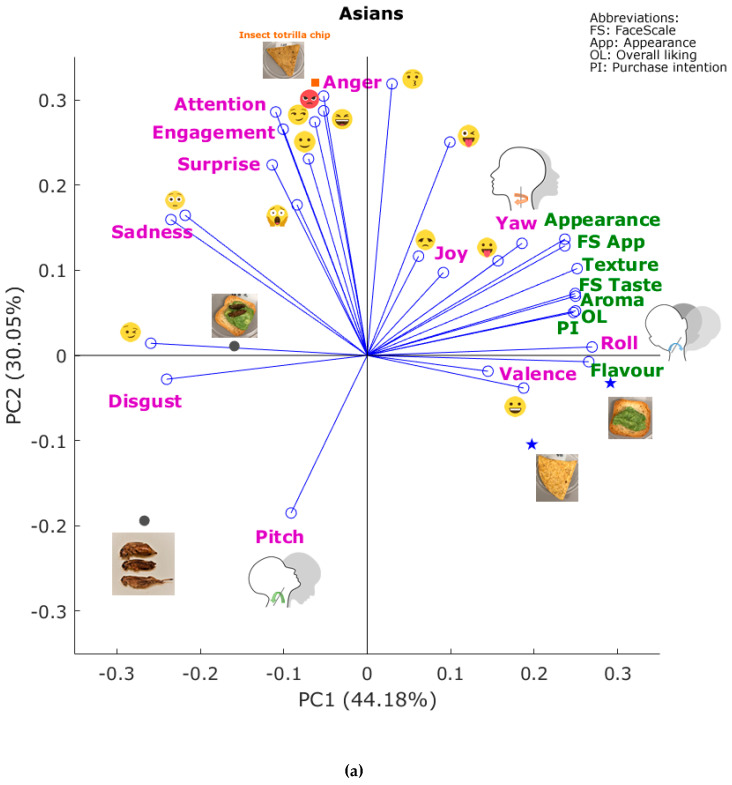

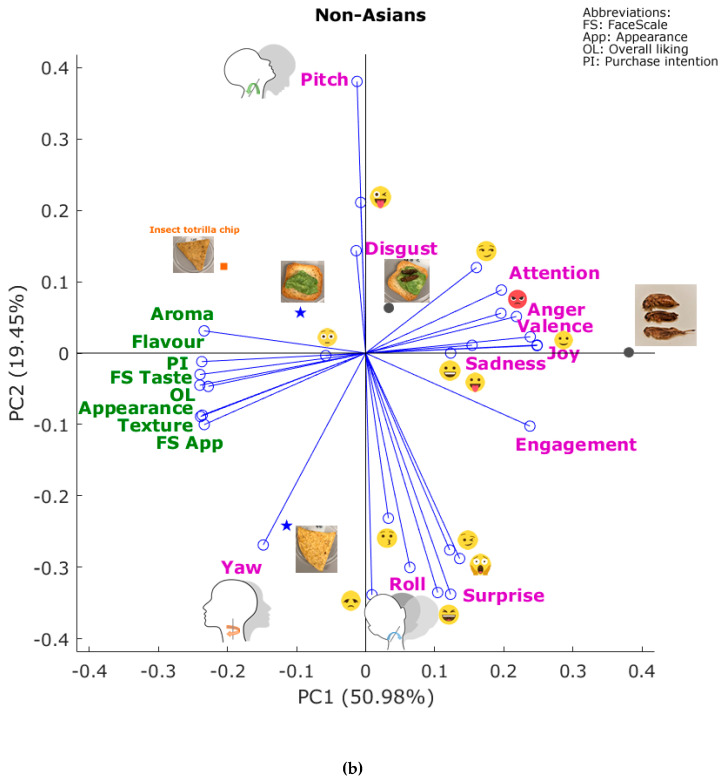
Principal components analysis for the biometric and quantitative self-reported responses for (**a**) Asians and (**b**) non-Asians. Abbreviations: PC1 and PC2: principal components one and two.

Figure 2a shows the PCA from Asians, which explains 74.23% of the total data variability (PC1 = 44.18%; PC2: 30.05%). According to the factor loadings (FL), the principal component one (PC1) was mainly represented by roll (FL = 0.27), flavor (FL = 0.27), and texture (FL = 0.25) on the positive side of the axis, and by winking face 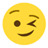 (FL = −0.26), disgust (FL = −0.24), and sadness (FL = −0.24) on the negative side. On the other side, PC2 was mainly represented by kissing 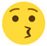 (FL = 0.32), rage 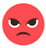 (FL = 0.30), and anger (FL = 0.30) on the positive side, and pitch 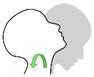 (FL = −0.19) on the negative side of the axis. It can be observed that both control samples (avocado toast and tortilla chip) were associated with a higher liking of flavor, purchase intention, overall liking, valence, smiley face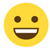, and roll 
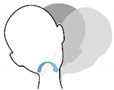
, while the tortilla chip made with cricket flour was associated with emotions and emojis such as anger, rage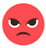, smirk 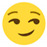, and laughing 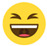. On the other hand, the avocado toast with crickets was associated with winking face 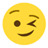, disgust, sadness, and flushed face 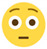, while the whole crickets were associated with disgust and pitch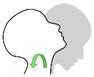.

Figure 2b shows the PCA for non-Asians, which explained 70.43% of the total data variability (PC1 = 50.98%; PC2 = 19.45%). The PC1 was mainly represented on the positive side of the axis by joy (FL = 0.25), smiley 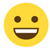 (FL = 0.25), engagement (FL = 0.24), and relaxed 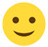 (FL = 0.24), and on the negative side by the self-reported responses (FL = 0.24) appearance, texture, flavor, overall liking, and purchase intention. The PC2 was mainly represented by pitch 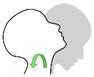 (FL = 0.38) and stuck out tongue with winking eye 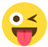 (FL = 0.21) on the positive side of the axis, and by surprise (FL = −0.34), laughing 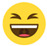 (FL = −0.34), and disappointed 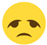 (FL = −0.34) on the negative side. The control sample of the tortilla chip was associated with yaw 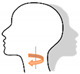, and disappointed 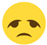, while the control sample of avocado toast and tortilla chip with cricket flour were mainly linked with the liking of aroma and flavor, as well as disgust. On the other hand, the avocado toast with crickets was associated with pitch 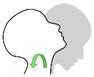, stuck out tongue with winking eye 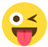, disgust, and smirk 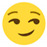, while the whole crickets were linked with joy, valence, relaxed 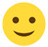, anger, rage 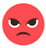, engagement, and attention.

#### 3.2.2. Correlation Analysis

**Figure 3:**
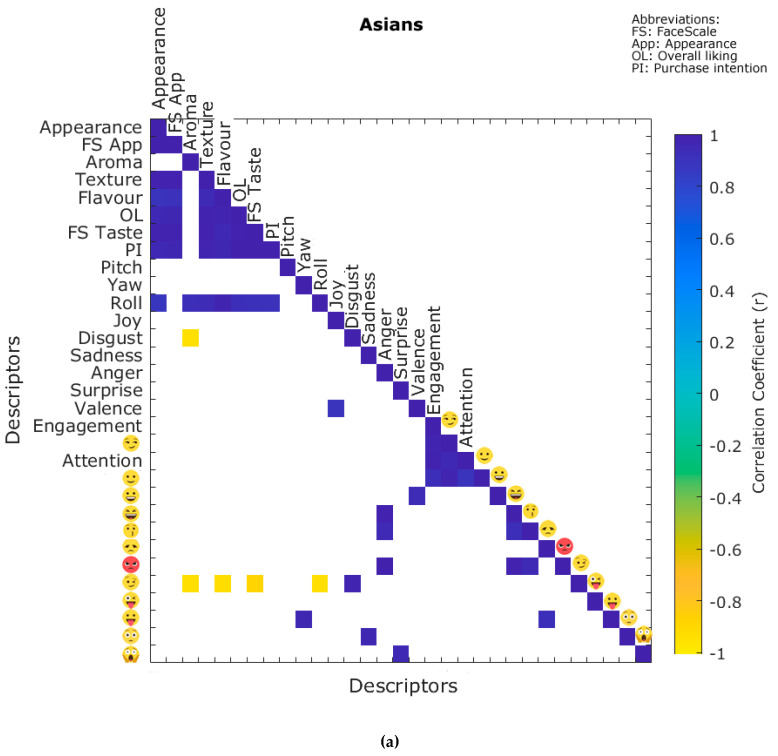

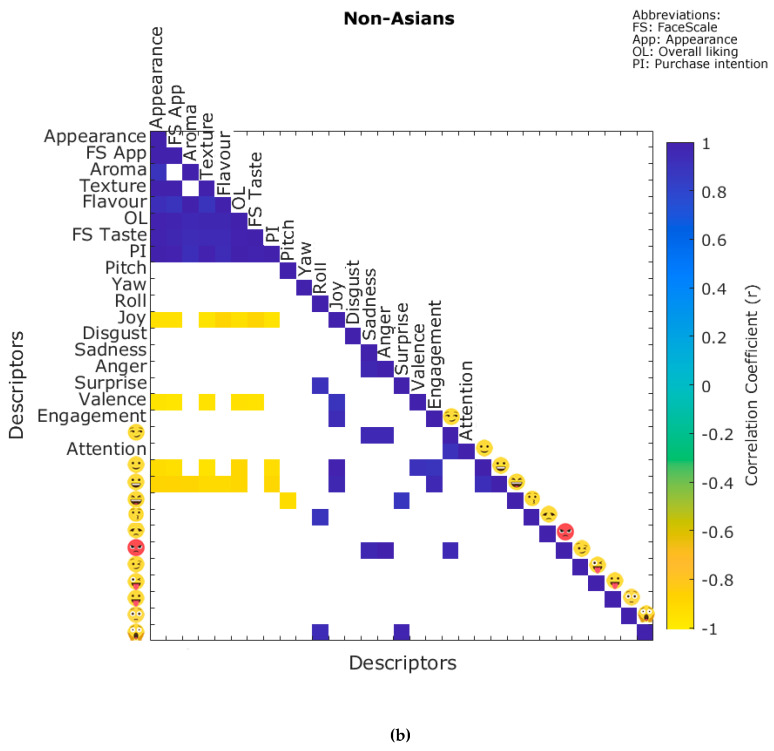
Matrices showing the significant (*p* ≤ 0.05) correlations between the quantitative self-reported and biometric emotional responses for (**a**) Asians and (**b**) non-Asians. Color bar: blue side depicts the positive correlations, while the yellow side represents the negative correlations; likewise, darker blue and yellow denote higher correlations.

Figure 3a shows the significant correlations (*p* ≤ 0.05) between the quantitative self-reported and biometric emotional responses for Asians. It can be observed that roll was positively correlated with the liking of appearance (correlation coefficient: *r* = 0.88), aroma (*r* = 0.92), texture (*r* = 0.93), flavor (*r* = 0.98), overall liking (*r* = 0.92), FS Taste (*r* = 0.91), and purchase intention (*r* = 0.91). The liking of aroma was negatively correlated with winking face 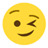 (*r* = −0.94), and disgust (*r* = −0.95), while the liking of flavor had a negative correlation with winking face 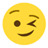 (*r* = 0.93), and the latter with FS Taste (*r* = −0.88). On the other hand, Figure 3b shows the significant correlations (*p* ≤ 0.05) found for non-Asians. It can be observed that joy had a negative correlation with the liking of appearance (*r* = −0.94), FS App (*r* = −0.95), the liking of texture (*r* = −0.97), flavor (*r* = −0.89), overall liking (*r* = −0.94), FS Taste (*r* = −0.89), and purchase intention (*r* = −0.96). Valence had a negative correlation with appearance liking (*r* = −0.97), FS App (*r* = −0.97), texture liking (*r* = −0.96), overall liking (*r* = −0.95), and FS Taste (*r* = −0.96). Similarly, relaxed 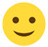 was negatively correlated with appearance liking (*r* = −0.92), FS App (*r* = −0.94), texture liking (*r* = −0.96), overall liking (*r* = −0.90), and purchase intention (*r* = −0.93). Smiley has a negative correlation with appearance liking (*r* = −0.89), FS App (*r* = −0.88), liking of aroma (*r* = −0.89), texture (*r* = −0.91), flavor (*r* = 0.92), overall liking (*r* = −0.90), and purchase intention (*r* = −0.90).

#### 3.2.3. Multiple Factor Analysis

**Figure 4:**
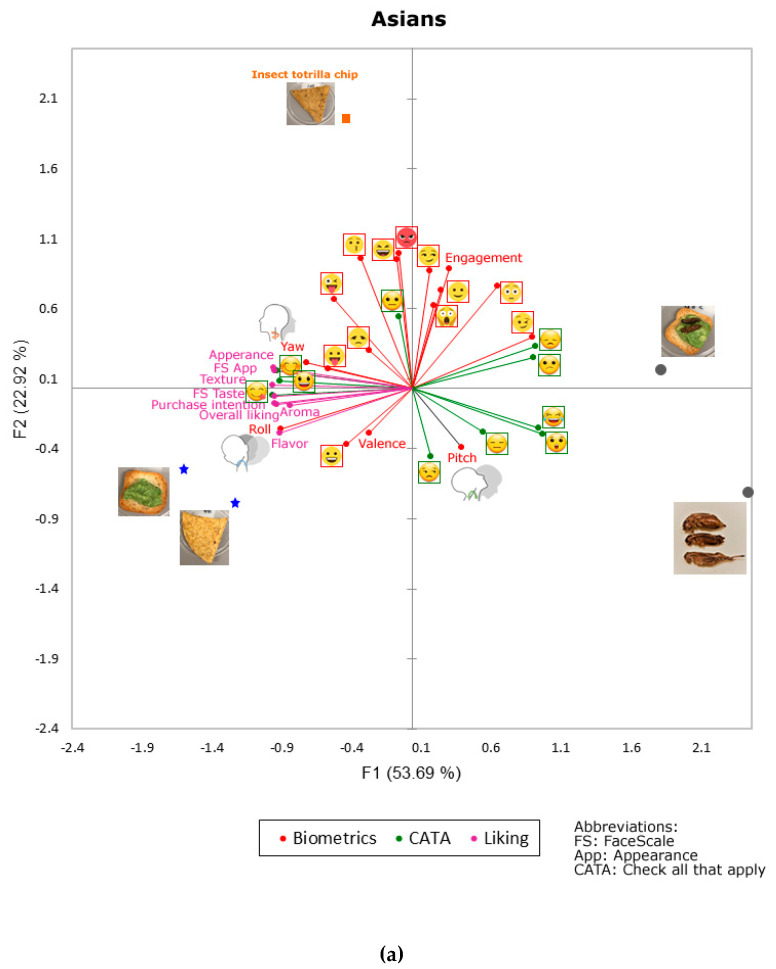

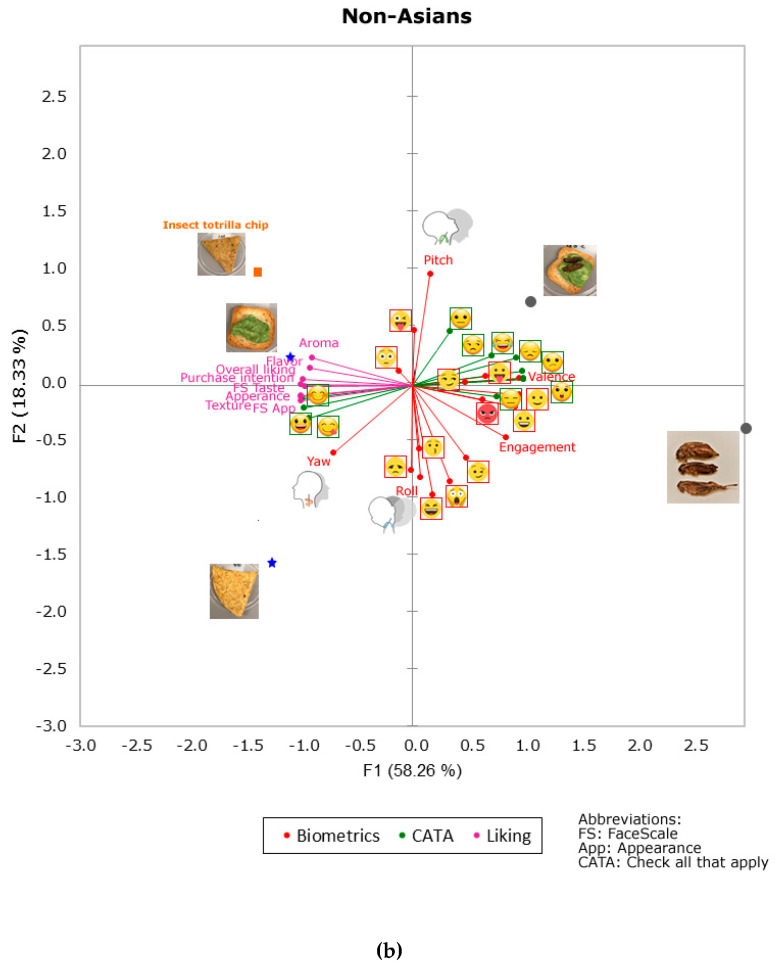
Multiple factor analysis for the biometric, frequencies (check all that apply: CATA) and quantitative self-reported (Liking) responses for (**a**) Asians and (**b**) non-Asians. Abbreviations: F1 and F2: factors one and two.

Figure 4a shows the MFA of Asian consumers, which explained a total of 76.61% (Factor 1: F1 = 53.69%; Factor 2: F2 = 22.92%). The F1 was mainly represented by surprised 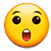 (FL = 0.93), and laughing 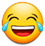 (FL = 0.91) on the positive side of the axis, and by savoring 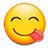 (FL = −1.00) and appearance liking (FL = −0.99) on the negative side. On the other hand, F2 was mainly represented on the positive side of the axis by rage 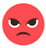 (FL = 0.97) and kissing 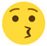 (FL = 0.93), and on the negative side by unamused 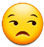 (FL = −0.49) and pitch 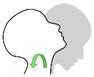 (FL = −0.42). It can be observed that the control samples (tortilla chip and avocado toast) were associated mainly with self-reported responses such as the liking of flavor, aroma, overall liking, and purchase intention, as well as some subconscious responses such as roll 
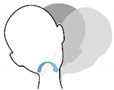
, smiley 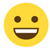, and valence. The tortilla chip made with cricket flour was associated with emojis measured with biometrics such as kissing 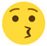, laughing 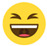, rage 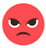, and stuck out tongue with winking eye 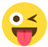, and with neutral 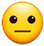 from the CATA test. On the other hand, the avocado toast with crickets was associated with emojis from the CATA test, such as disappointed 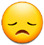, confused 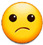, and winking face 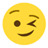 from the biometrics. The whole crickets were linked to emojis from the CATA test, such as laughing 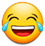, surprised 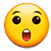, expressionless 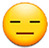, and pitch 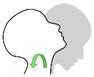.

Figure 4b shows the MFA of non-Asian consumers, which explained a total of 76.59% (F1 = 58.26%; F2 = 18.33%). Based on the FL, the F1 was mainly represented by surprised 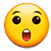 (FL = 0.99), disappointed 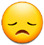 (FL = 0.99), and confused 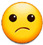 (FL = 0.99) on the positive side of the axis, and by texture liking (FL = −1.00), happy 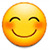 (FL = −0.99), liking of appearance (FL = −0.99), FS App (FL = −0.99), and purchase intention (FL = −0.99) on the negative side. On the other hand, F2 was represented by pitch 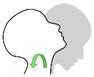 (FL = 0.97) on the positive side of the axis, and by laughing 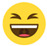 (FL = −0.95) and scared 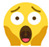 (FL = −0.84) on the negative side. The control sample of the tortilla chip was mainly associated with head movements such as yaw 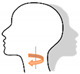 and roll 
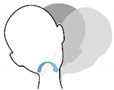
 and disappointed 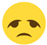. The control sample of the avocado toast and the tortilla chip made with cricket flour were associated with self-reported responses such as the liking of aroma, flavor, overall liking, and purchase intention as well as flushed 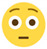. The avocado toast with crickets was mainly associated with emojis from the CATA test, such as laughing 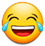, disappointed 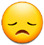, unamused 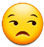, confused 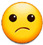, and neutral 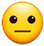, and with biometric responses such as valence, stuck out tongue 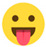, and smirk 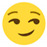. On the other hand, the whole crickets were linked to the biometric responses such as engagement, rage 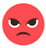, winking face 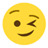, and relaxed 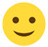, and with emojis from the CATA test such as surprised 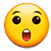 and expressionless 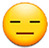.

### 3.3. Machine Learning Modeling

**Figure 5:**
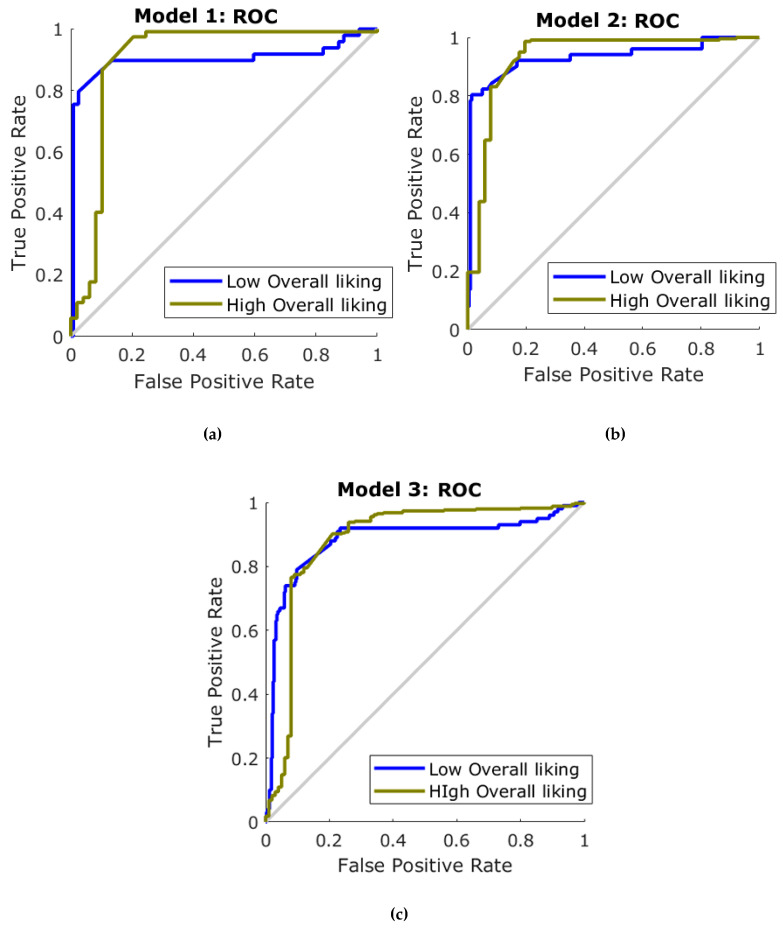
Receiver operating characteristics (ROC) curves for the three artificial neural network models for (**a**) Model 1: Asians, (**b**) Model 2: non-Asians, and (**c**) Model 3: general (Asians + non-Asians).

In Table 5, it can be observed that Model 1, developed with the results from the Asian participants, had a 92% accuracy to classify the samples into high and low liking using only the emotional responses from biometrics as inputs. Model 2 for non-Asians had a higher overall accuracy (94%) compared to the Asians, and with higher accuracy also in the training stage (non-Asians: 76%; Asians: 71%). On the other hand, Model 3, which was developed as a general model with data from all the participants (Asians and non-Asians), presented an overall accuracy of 89%. The three models had a lower MSE value for the training stage compared to testing, which indicates that there was no overfitting. Figure 5 shows the receiver operating characteristics (ROC) curves, which depict the performance based on the specificity (false positive rate) and sensitivity (true positive rate) of each model and classification group.

## 4. Discussion

In general, for this study, consumer perception had higher scores for self-reported information for the control samples, which did not contain insects, and the lowest scores for full insects, as expected. These scores were similar to the sample with insects as an ingredient, in which no parts of insects were visible, which is in accordance with previous literature [1,14,31]. However, mid-range liking, FS Taste, and purchase intention were obtained for visible insects mixed with more familiar products, such as toast and avocado mash (Table S1).

When combining self-reported information with the subconscious and emotional responses through the PCAs (Figure 2), it was found that high and similar variability of data was explained for both the Asian and non-Asian participants (>70%). For the Asians (Figure 2a), more negative emotional responses were associated with insect-based samples compared to the controls, with a clear separation in the vertical plane (PC1). For the non-Asians (Figure 2b), a similar separation was observed for the samples with visible insects, with a difference in the insect tortilla chip. These results may seem contrasting with the self-reported data; however, the subconscious responses are related to the first impression of the samples for visible, aroma, and taste responses from consumers, which are spontaneous and more complex when tasting food and beverage products of any kind [32,33,34]. Similar relationships were found using the correlation matrix analysis (Figure 3) and the further MFA combining the self-reported data, biometrics, and CATA.

A deeper understanding of consumer acceptability, liking, and intention to purchase new insect-based food products is extremely important, since around 95% of new food products may fail in the market without proper assessment [35,36]. Predictive modeling incorporating cultural backgrounds may offer more information and the possibility of automation for the decision-making process or product variation when developing new insect-based food products. Recent research has been based on automatic estimations of liking based on facial expression dynamics, especially for infants, since self-reported data may not be easily obtained [37,38]. However, research on automatic assessments based on biometrics is rarer [15,28,32].

By using ML modeling considering the separation of cultural background, it was possible to obtain high accuracy (≥92%) in the prediction of liking based on the biometrics from Asians (Table 5; Figure 5a) and non-Asians (Table 5; Figure 5b) using the Bayesian Regularization ANN algorithm. However, since ML using ANN is considered to be a robust method to detect patterns within data, the cultural distinction could be an internal feature of a general model, as shown in Table 5 and Figure 5c. This general model, with an accuracy of 89%, may be used to detect the liking levels of snacks containing insects as part of their ingredients or as whole insects. Further research is required to test and model the biometric responses from a wider variety of insect-based food or beverages.

A quicker analysis for consumer acceptability, liking, and intent to purchase can be achieved as a first approach by presenting images of dishes prepared with insects through the BioSensory app to obtain biometrics from panelists. It has been previously shown for packaging assessments that presenting images of the packaging, and the physical packaging samples resulted in non–significant statistical differences in their appreciation by panelists in a sensory trial [39]. Similarly, for beer tasting panelists looking at videos of beer pouring gave similar levels of liking and general appreciation compared to those that tasted the same beers [15,40]. The applicability for the assessment of images of insect-based dishes is supported by the data presented in this study, especially related to the statistically significant correlations between the appearance and overall liking for both cultures (Figure 3a,b) and the higher variability of the data explained by PC1 for both Asians and non-Asians (Figure 2a,b).

The advantages of using both the self-reported and biometric responses rely on the fact that they allow us to obtain the first and subconscious reaction that consumers have towards the product they are assessing as well as the way they may modify their responses after the thinking process. This aids in a deeper assessment of consumers’ behavior and acceptability towards food and beverages to understand the target market and develop products according to their needs. The results from Asians and non-Asians were statistically analyzed separately due to the cultural differences that may be related to the expression of emotional responses. This was also the main reason for developing ML models for each culture and a general model considering both cultures. However, despite that Model 3 (general model) had a slightly lower accuracy, it was shown that ANN is able to accurately find patterns among data that may be related to differences in responses from different cultural backgrounds to predict liking regardless.

## 5. Conclusions

The introduction of insect-based food and beverage products in the market may be a viable alternative for sustainable and nutritional sources of protein. However, the neophobia associated with these products is based on the cultural background and lack of familiarity. More studies are required to break misconceptions and insect-based phobia, which requires the study of complex interactions between the response of consumers to insect-based food products due to visual first impressions, aroma, taste, emotional responses, and cultural background. The implementation of biometrics and machine learning modeling could provide deeper insights regarding these complex interactions and the liking/purchase intention of insect-based food products. Computer vision and machine learning should be further explored and researched as the basis for the development of an artificial intelligence approach to assess new food products and potential success in the market. 

## Figures and Tables

**Table 1:** Image and description of samples used in the sensory session.

Sample Image	Sample Description
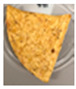	Tortilla chip with cornflour (Control)
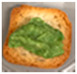	Toast with avocado (Control)
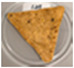	Tortilla chip with corn and cricket flour
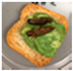	Toast with avocado and crickets
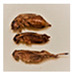	Roasted crickets

**Table 2:** Descriptors and scale used in the questionnaire to acquire self-reported responses. Questionnaires were uploaded in the BioSensory app, including sample numbers, descriptors, scales, and emoticons.

Descriptor	Scale	Anchors	Label
Appearance	15 cm non-structured	Dislike extremely-Neither like nor dislike-Like extremely	Appearance
Appearance	FaceScale (0–100)	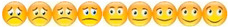	FS App
Aroma	15 cm non-structured	Dislike extremely-Neither like nor dislike-Like extremely	Aroma
Texture	15 cm non-structured	Dislike extremely-Neither like nor dislike-Like extremely	Texture
Flavor	15 cm non-structured	Dislike extremely-Neither like nor dislike-Like extremely	Flavor
Overall liking	15 cm non-structured	Dislike extremely-Neither like nor dislike-Like extremely	OL
Tasting	FaceScale (0–100)	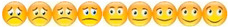	FS Taste
Purchase intention	15 cm non-structured	Dislike extremely-Neither like nor dislike-Like extremely	PI

**Table 3:** Emojis used for the check all that apply questions for the sensory test using the BioSensory app.

Emoji	Meaning	Emoji	Meaning
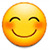	Happy	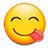	Savoring
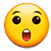	Surprised	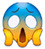	Scared
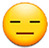	Expressionless	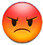	Angry
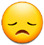	Disappointed	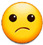	Confused
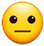	Neutral	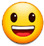	Joy
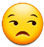	Unamused	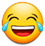	Laughing

**Table 4:** Facial expressions and emotion-related parameters obtained from the biometric video analysis (Affectiva app).

Parameter	Label	Parameter	Label	Parameter	Label
**Joy**	Joy	**Winking face**	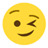	**Lip Corner Depressor**	LCD
**Disgust**	Disgust	**Rage**	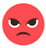	**Lip Press**	LPr
**Sadness**	Sadness	**Smirk**	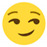	**Lip Suck**	LS
**Surprise**	Surprise	**Disappointed**	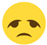	**Mouth Open**	MO
**Anger**	Anger	**Scared**	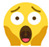	**Smirk Facial Expression**	SmirkFE
**Fear**	Fear	**Stuck out tongue with winking eye**	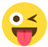	**Eye Closure**	EC
**Contempt**	Contempt	**Laughing**	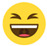	**Eye Widen**	EW
**Valence**	Valence	**Kissing**	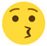	**Cheek Raise**	CR
**Smile**	Smile	**Inner Brow Raise**	IBR	**Lid Tighten**	LT
**Engagement**	Engagement	**Brow Rise**	BR	**Dimpler**	Dimpler
**Attention**	Attention	**Brow Furrow**	BF	**Lip Stretch**	LSt
**Smiley**	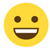	**Nose Wrinkle**	NW	**Jaw Drop**	JD
**Relaxed**	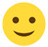	**Upper Lip Rise**	ULR	**Pitch**	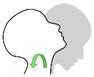
**Stuck out tongue**	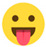	**Chin Raise**	CR	**Yaw**	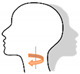
**Flushed**	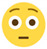	**Lip Pucker**	LP	**Roll**	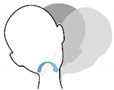

**Table 5:** Statistical data from the three artificial neural network models. Performance shown is based on the mean squared error (MSE)**.**

Stage	Samples × Participants	Accuracy	Error	Performance (MSE)
Model 1: Asians				
Training	134	97%	3%	0.03
Testing	34	71%	29%	0.24
Overall	168	92%	8%	-
Model 2: Non-Asians				
Training	216	99%	1%	0.01
Testing	54	76%	24%	0.20
Overall	270	94%	6%	-
Model 3: General				
Training	350	97%	3%	0.03
Testing	88	71%	29%	0.25
Overall	438	89%	11%	-

## Supplementary Materials

The following are available online at https://www.mdpi.com/2304-8158/9/7/903/s1: Table S1: Means and standard error of the self-reported responses for each sample per culture. Different letters denote significant differences based on ANOVA and Fisher’s least significant difference post hoc test (α = 0.05). Table S2: Means and standard error of the statistically significant biometric responses for each sample per culture. Different letters denote significant differences based on ANOVA and Fisher’s least significant difference post hoc test (α = 0.05).
