# Supplementary material for: Non-Invasive Biometrics and Machine Learning Modeling to Obtain Sensory and Emotional Responses from Panelists during Entomophagy
Source: Foods. 2020 Jul 9;9(7):903. doi: 10.3390/foods9070903 (PMC7404998; doi:10.3390/foods9070903)
Supplement: Supplementary file 1 [file foods-09-00903-s001.pdf]

Table S1. Means and standard error of the self-reported responses for each sample per culture. Different letters denote significant differences based on ANOVA and Fishers least significant difference *post hoc* test (α = 0.05). .

Culture*Sample/ Descriptors	Appearance	FaceScale Appearance	Aroma	Texture	Flavor	Overall liking	FaceScale Taste	Purchase intention
Asian* 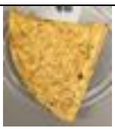	11.14ab	77.29ab	10.45abc	11.40ab	11.25ab	11.35ab	78.18abc	9.94ab
	±0.55	±3.86	±0.57	±0.59	±0.53	±0.55	±3.70	±0.84
Non-Asian* 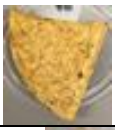	12.00a	82.04a	10.09bc	12.17a	11.76a	11.61a	79.67ab	10.58a
	±0.35	±2.18	±0.40	±0.38	±0.34	±0.33	±2.28	±0.52
Asian* 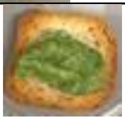	11.17ab	75.33ab	11.37a	11.60ab	11.33ab	11.18abc	75.49abc	9.27abc
	±0.61	±3.65	±0.48	±0.61	±0.62	±0.70	±4.51	±0.85
Non-Asian* 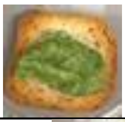	11.69ab	77.78ab	11.09ab	11.75a	12.23a	12.22a	84.02a	10.95a
	±0.30	±2.22	±0.34	±0.34	±0.31	±0.31	±2.02	±0.49
Asian* 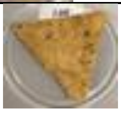	10.54b	71.85b	9.16c	10.85ab	**9.91bc**	**10.14bc**	70.27bc	**8.16bc**
	±0.54	±3.09	±0.55	±0.66	±0.70	±0.66	±4.02	±0.85
Non-Asian* 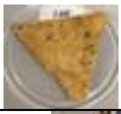	11.15ab	77.06ab	10.36abc	12.01a	**11.64a**	**11.63a**	77.76abc	**10.66a**
	±0.39	±2.34	±0.42	±0.36	±0.37	±0.38	±2.37	±0.49
Asian* 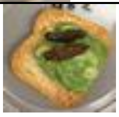	5.94cd	43.77cd	9.20c	9.20cd	**9.09cd**	**7.95d**	**57.27d**	**5.56d**
	±0.76	±5.14	±0.60	±0.73	±0.75	±0.81	±5.31	±0.92
Non-Asian* 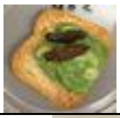	7.23c	51.54c	9.86c	10.16bc	**11.20ab**	**9.83c**	**69.78c**	**7.81c**
	±0.52	±3.21	±0.37	±0.46	±0.37	±0.43	±3.00	±0.56
Asian* 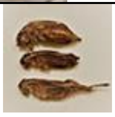	4.50de	36.09d	6.54d	8.67d	9.01cd	8.14d	54.97d	5.62d
	±0.66	±4.99	±0.54	±0.78	±0.76	±0.82	±5.44	±0.80
Non-Asian* 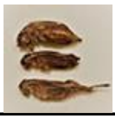	3.81e	35.65d	6.84d	8.93cd	8.16d	7.48d	59.36d	5.38d
	±0.41	±2.96	±0.34	±0.51	±0.42	±0.43	±3.20	±0.56

Table S2. Means and standard error of the statistically significant biometric responses for each sample per culture. Different letters denote significant differences based on ANOVA and Fishers least significant difference *post hoc* test ($\alpha = 0.05$).

**Culture*Sample/ Descriptors**	**Roll**	**Joy**	**Engagement**	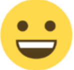	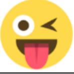
Asian* 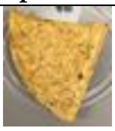	-0.51ab	4.10abc	15.14abc	1.74ab	2.11c
	±0.49	±1.59	±3.04	±0.74	±0.08
Non-Asian* 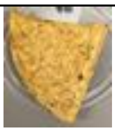	0.01a	2.01c	13.39bc	0.43bc	2.07c
	±0.47	±0.71	±1.28	±0.18	±0.04
Asian* 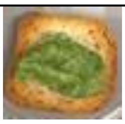	0.21a	6.68a	15.88ab	2.57a	2.40bc
	±0.70	±2.10	±2.45	±0.96	±0.12
Non-Asian* 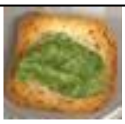	-0.03a	2.35bc	13.21bc	0.59bc	2.27abc
	±0.66	±0.70	±1.51	±0.26	±0.11
Asian* 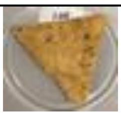	-1.66abc	4.80abc	18.84ab	1.22abc	2.47a
	±0.63	±2.07	±3.23	±0.52	±0.16
Non-Asian* 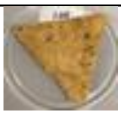	-0.48ab	1.18c	9.41c	0.29c	2.10c
	±0.60	±0.45	±1.10	±0.14	±0.06
Asian* 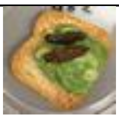	-2.25bc	7.05a	18.66ab	2.05a	2.11c
	±0.94	±2.06	±2.39	±0.74	±0.07
Non-Asian* 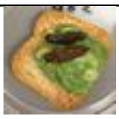	-0.20a	3.61abc	13.40bc	0.64bc	2.21bc
	±0.94	±2.06	±2.39	±0.74	±0.07
Asian* 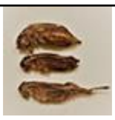	-2.77c	2.90abc	15.39ab	1.06abc	2.10c
	±1.06	±0.92	±2.17	±0.54	±0.09
Non-Asian* 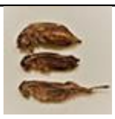	-0.11a	5.49ab	18.96a	1.31abc	2.12c
	±0.45	±1.53	±2.19	±0.49	±0.04
